# Association between Social Participation and Remaining Teeth and Urban–Rural Difference among Older Adults in China

**DOI:** 10.3390/ijerph20021283

**Published:** 2023-01-10

**Authors:** Le Yang, Dan Guo, Jiaming Zheng, Yuting Guo, Zeyuan Li

**Affiliations:** School of Management, Shanxi Medical University, 56 Xinjian South Road, Taiyuan 030001, China

**Keywords:** social participation, oral health, older adults, urban–rural difference, China

## Abstract

Oral health is an important part of older adults’ general health. The study examined the association between social participation (formal and informal) and remaining teeth and the urban–rural difference based on a national survey of older adults in China. The data of older adults were extracted from the Chinese Longitudinal Healthy Longevity Survey (CLHLS) and analyzed. A total of 11,948 respondents were ultimately involved, including 6836 urban respondents and 5112 rural respondents. Informal social participation and formal social participation were used to assess social participation. The number of remaining natural teeth was measured. Social participation was significantly associated with remaining teeth among older adults, after adjusting for confounders, a one-level increase in the informal social participation was associated with a decrease in natural teeth by 0.152 (95% CI = −0.274; −0.030) and a one-level increase in the formal social participation was associated with a decrease in natural teeth by 0.370 (95% CI = −0.585; −0.156). In addition, the association between social participation (formal and informal) and remaining teeth was observed among urban older adults, but not rural older adults. A high level of social participation may effectively decrease the risk of oral-health problems for the Chinese older adults. The findings suggest recommendations for an older adults-targeted policy and the practice of oral-health promotion. However, the urban–rural difference should be taken into full consideration in social-participation-driven oral-health promotion interventions.

## 1. Background

Since China has become an aging society, the number of older adults is increasing. The government and the public are increasingly concerned about the quality of life and well-being of older adults. Oral health is an important part of the general health of older adults [1,2]. The Fourth National Epidemiological Survey of Oral Health reported that in 2016, 4.5% of older adults aged 65 to 74 were edentate in China, and the oral health of older adults is relatively poor. Oral health not only affects chewing, pronunciation, and the image of older adults, but is also closely related to cardiovascular disease, respiratory disease, diabetes, digestive-system diseases and other systemic diseases [3,4,5]. The oral health status of older adults and its influencing factors has become a major concern of scholars all over the world. A large amount of research has explored risk factors for older adults’ oral health, ranging from eating habits, oral-health behaviors, food intake, socioeconomic status, and acquired systemic conditions [2,6,7]. Amongst these factors, social participation has been attracting attention for its assumed positive effect on older adults’ health.

The positive impact of social participation on older adults’ health has been long acknowledged [8,9,10]. Many studies have reported that social participation had a protective effect on health outcomes such as cognitive decline [11], functional decline [12], and depressive symptoms [13]. Social participation is assumed to influence health through two potential ways (i.e., direct effects and buffering effects), as a direct effect, social participation is related to more frequent and regular physical activity and interaction, which contribute to the improved physical-health status and reduce the risk of mental illness [12,14]. Social participation can act as a buffer by providing access to more available social support and additional resources to enhance suitable coping mechanisms for stressful events and health-promotion mechanism based on information access and behavior intervention [15,16].

Recently, a growing body of research has examined the association between social participation and oral health and how social participation may benefit oral health. Previous studies have showed lower levels of social participation were associated with being edentate [17,18] or periodontitis [19]. Oral health is strongly linked to social and behavioral factors [20]. In the context of different social cultures and food habits, the association between social participation and oral health may be different. For Asian people, Takeuchi et al. (2013) reported that a better dental-health status was significantly associated with higher social-participation rates, but no significant association was found between frequent participation in neighborhood communities and dental health [21]. Another study revealed that better oral health had a significant association with higher levels of social activities, which was partially mediated by decreased depressive symptoms [22]. However, the current literature on the association between social participation and oral health among older adults is still underdeveloped in China.

Concerning the protective role of social participation in oral health, the types of social participation and urban–rural difference in the effects of social participation have not received enough attention till now. Social participation can be divided into formal social participation and informal social participation: formal social participation refers to structured activities, such as involvement in organized social activities, while informal social participation involves more casual forms of social contact, such as interaction with friends [23,24]. Formal social participation and informal social participation may have different effects and act in different ways on quality of life and well-being among older adults [25,26,27]. However, the two key dimensions do not receive enough attention in most studies on social participation and oral health. In addition, previous research showed the urban–rural difference in oral health in China, such as the number of remaining teeth, dental healthcare utilization, and root caries [28,29,30]. Meanwhile, the social participation of rural older people may be different to that of urban older people. Compared to those who live in urban areas, rural older people were less socially active [31,32]. Thus, urban–rural difference should receive more discussion in research on the social participation–oral health association among older adults.

Given the data extracted from Chinese Longitudinal Healthy Longevity Survey (CLHLS), the study examines the association between social participation (formal and informal) and remaining teeth and the urban–rural difference based on a national survey of older adults in China. It is hypothesized that both high levels of formal and informal social participation are more likely to be associated with better oral health (i.e., more remaining teeth) among Chinese older adults. In addition, there may be differences between rural and urban older adults in the social participation–remaining teeth association.

## 2. Methods

### 2.1. Study Design and Population

CLHLS was conducted on a randomly selected sample covering 23 provinces [33]. In 2017−2018, CLHLS conducted national surveys with 15,874 individuals (wave 8). From this study, there were 3926 respondents excluded, in total. Those who were under 65 years old (*n* = 102) or lacked self-reported teeth (*n* = 217) were excluded. The respondents who did not answer all related questions were regarded as unreliable and were excluded from the analysis; among them, 135 individuals and 105 individuals lacked formal and informal social-participation-related information, respectively; 2883 individuals did not have complete sociodemographic data; and 484 individuals did not provide health-related information. A total of 11,948 respondents were ultimately analyzed, with 6836 urban respondents and 5112 rural respondents.

### 2.2. Measures

#### 2.2.1. Remaining Teeth

The number of natural teeth was measured based on the responses collected through the question of “How many natural teeth do you have (excluding dentures)?”.

#### 2.2.2. Social Participation

According to the measurement of social participation in prior studies [23,34,35,36,37] and the data from CLHLS, social participation was measured as follows. (1) Question about formal social participation: Do you take part in social activities (organized) regularly? The answer options ranged from 1 to 5, which meant “almost every day”, “at least once a week”, “at least once a month”, “sometimes”, and “never”. (2) Question about informal social participation: Do you visit and interact with friends regularly? The answer options ranged from 1 to 5, which meant “almost every day”, “at least once a week”, “at least once a month”, “sometimes”, and “never”.

#### 2.2.3. Covariates

Demographic and socioeconomic characteristics and health behaviors and health condition were involved in the analysis as potential confounders. Demographic and socioeconomic characteristics include age, gender, education background (assessed as years of schooling), marital status, and annual household income. Years of schooling was divided into 0 (uneducated), 1–6 years (primary school), 7–12 years (middle school and high school), and ≥13 years (college or above). Marital status was categorized as “married and living with spouse, separate, divorced, widowed, or never married”. Household annual income was divided into low (≤12,000 yuan), medium (12,000−55,000 yuan), and high (>55,000 yuan). Health behaviors include current smoking (yes vs. no), current alcohol drinking (yes vs. no), and sleep duration (<7 h, 7–9 h, and >9 h). For health condition, the study selected body mass index (BMI), activities of daily living (ADL), cognitive function, and noncommunicable disease (NCD) as potential confounders. BMI category was calculated using the standard weight status categories from WHO reference, including underweight (<18.5 kg/m^2^), normal weight (18.5–24.9 kg/m^2^), overweight (25–29.9 kg/m^2^), and obesity (≥30 kg/m^2^) [38]. ADL was assessed based on six basic daily living items, with lower scores indicating better functional independence [39]. Cognitive function was measured using the Chinese version of Mini-Mental State Examination (MMSE) [40]. NCD was treated as a binary outcome (yes vs. no).

### 2.3. Statistical Analysis

This study was conducted to explore the association of social participation and number of remaining teeth as well as its urban–rural difference in the older adults. The continuous variables were described using mean and standard deviation (SD), and the categorical variables were presented by number and proportion (%). The multivariable multilevel linear regression analyses were performed to explore the association between social participation and number of remaining teeth among older adults. Model 1 was crude without any confounders, and Model 2 was adjusted for demographic and socioeconomic characteristics. In Model 3, we adjusted for health behaviors and health condition based on the confounding variables in Model 2. Collinearity diagnostics were also conducted in linear regressions in order to assess the potential for regression coefficient instability. The tests for linear regression show a variance inflation factor (VIF) ranging from 1.070 to 2.201, which is lower than the recommended cut-off threshold of 10. To examine urban–rural differences in the association of social participation and remaining teeth, we performed stratified analyses by residence group, adjusting for all confounders.

All statistical analyses were performed by SPSS ver. 24.0 (IBM, Armonk, NY, USA). The significance level was at *p*-value < 0.05.

## 3. Results

The characteristics of the older adults are summarized in Table 1. About 43.0% of the participants were men and 57.0% were women, and the average age was 85.48 ± 11.87 years old. A higher percentage of the older population with a high household annual income was reported in urban respondents (*p* < 0.001). A total of 25.7% of the urban older adults had a middle school degree or above, and while of those living in rural areas this value was 9.3%. Meanwhile, rural older adults tended to show a higher proportion of smoking and drinking than urban older adults, although there was no statistical significance regarding drinking. In addition, for those who were overweighted or obese, the proportion of urban respondents was relatively higher (24.8%). The prevalence of NCD in rural older adults was higher than that in urban older adults (*p* < 0.001). The mean scores of ADL and MMSE in urban respondents were relatively higher among those rural respondents (*p* < 0.001).

A total of 45.4% of the older adults had never participated in informal social activity and 86.4% of the older adults had never participated in formal social activity. A higher proportion of rural respondents reported informal social participation almost everyday (28.0%), but the proportion of rural residents who never participated in formal social activities was also very high (91.4%). The mean number of natural teeth of respondents was 9.48 (SD = 10.40), and urban older adults reported more natural teeth than rural older adults (*p* < 0.001).

Table 2 shows the multiple linear regression analysis results of the association between social participation and remaining teeth among older adults. Across all models, a significant association was found both between informal social participation and formal social participation and remaining teeth. In Model 1, a one-level increase in informal social participation (i.e., lower informal social participation frequency) was associated with a decrease in natural teeth by 1.142 (95% CI = −1.271; −1.014), and a one-level increase in formal social participation (i.e., lower formal social participation frequency) was associated with a decrease in natural teeth by 1.672 (95% CI = −1.910; −1.434). After adjusting for confounders (gender, age, education background, marital status, household annual income, smoking, alcohol drinking, sleep duration, BMI, NCD, ADL, and cognitive function), the association were attenuated in Model 3. A one-level increase in informal social participation was associated with a decrease in natural teeth by 0.152 (95% CI = −0.274; −0.030) and a one-level increase in formal social participation was associated with a decrease in natural teeth by 0.370 (95% CI = −0.585; −0.156).

We further examined the association between social participation and remaining teeth by residence. As shown in Table 3, social participation, both of the formal and informal type, was significantly associated with remaining teeth among urban older adults, but not rural older adults. In urban older adults, a one-level increase in informal social participation (i.e., lower informal social participation frequency) was associated with a decrease in natural teeth by 0.244 (95% CI = −0.408; −0.081) and a one-level increase in formal social participation (i.e., lower formal social participation frequency) was associated with a decrease in natural teeth by 0.309 (95% CI = −0.566; −0.052) after adjusting for all confounders.

## 4. Discussion

Our findings suggest that social participation (formal and informal) has a significant relationship with remaining teeth among older adults, which was consistent with previous studies [17,21], although the association was modest.

*Formal social participation*: This study finds evidence that a low frequency of formal social participation is associated with a high risk of natural teeth decreasing among older adults. Formal social participation is greatly beneficial to the physical and psychological condition of older adults from a health-promotion perspective, but in this study, older adults reported low levels of formal social participation. Formal social participation could enhance the positive feelings of older adults [41] and protect them against depressive symptoms [42,43,44], which may help them to maintain an active lifestyle [45]. Some studies suggested that formal social participation could foster a stronger sense of identity, i.e., self-esteem, self-confidence, and self-efficacy, which improves health behaviors, such as good oral habits and dietary habits, and leads to positive psychological health [46,47]. In addition, older adults could obtain social support from formal social participation [48,49], increasing access to health-related information and health services, helping participants make better health and medical choices, and improving their attention to oral health.

*Informal social participation*: To our knowledge, there are few studies focusing on the association between informal social participation and remaining teeth among older adults, especially in China. The results indicate that informal social participation played an important role in the oral health of older adults. Informal social participation refers to the engagement of casual social activity with family and friends, which is more common than formal social participation in Chinese culture and tradition. A study of older adults in Japan found that dental-health behavior and stress are influenced mainly by close friends [50]. Facing significant psychological distress may lead to an increase in oral-health-related risk behaviors, such as smoking and/or the consumption of confectionary, which can lead to tooth loss [51,52]. Informal social participation can reduce the sense of solitude, decrease stress and make old adults more positive in life [53], and can also result in higher compliance with positive health behaviors [54]. However, the potential negative effect of informal social participation on health should not be neglected, such as a negative peer influence on health-risk behaviors (smoking, diets high in sugar) [17,55].

We observe that urban older adults benefited from social participation, both formal and informal, but not rural older adults. Urban residents have been found to have greater social participation and wider social interaction than rural residents, due to better transportation conditions, adequate essential activities, and good infrastructure [56,57]. A previous study claimed that individuals with relatively few close social ties may have benefited from formal social participation, while formal social participation among those with many social ties did not appear to be beneficial [58]. This seems not to be consistent with the results of this study; the reason for that may be the difference in objects and context. Older adults living in rural places are often idealized as possessing limited but stronger ties to their communities and retaining high-quality relationships with friends for decades [41,59]. Compared with participating in organized social activities, rural residents prefer unstructured social activities with their friends in China. It may also be attributed to the association between gender and social participation. Considering Chinese traditional social roles and the social responsibilities of different genders [60], women pay more attention to their relationships with family and friends, while men have more close relationships outside of their family, such as colleagues, groups, and organizations, etc. In this study, women respondents constituted a higher proportion both in rural and urban areas, which seems to partly explain the reasons for the findings. With the rapid development of the economy and society, a growing number of the young and middle-aged labor force have poured into cities to seek more employment opportunities, better education opportunities and higher incomes. For those women older adults who are still living in rural areas, their informal social participation could be greatly decreasing with age because they have fewer living friends and lower interaction frequency, with friends relocating or following their children to the city [61,62]. In addition, an insufficiency of oral health knowledge is quite common in Chinese rural residents [63]; the current social participation pattern (i.e., a high level of informal social participation and low level of formal social participation) could not help them to build good oral-health behaviors, improve oral-health-related information acquisition, or enhance oral-health-service utilization.

Several limitations should be noted of this study. First, the analysis was conducted based on a cross-sectional data; thus, no cause–effect relationships between social participation and remaining teeth among older adults could be identified. Second, the social-participation measurements in this study were limited due to a lack of data; therefore, the accuracy of our estimation of the association between social participation (formal and informal) and remaining teeth may be compromised. Last, this study found that social participation, both formal and informal type, was significantly associated with the number of natural teeth in urban older adults, but not in rural older adults, the underlying mechanisms were still notional. Despite these limitations, our findings contribute to current limited research on social participation and remaining teeth. Further study could examine social-participation interventions to improve the oral health of older adults and further explore the mechanism of urban–rural difference in the impact of social participation on oral health in different contexts.

## 5. Conclusions

Overall, this study suggests that policymakers need to give more careful consideration to social-participation interventions for oral-health promotion among older adults according to the significant association between social participation and remaining teeth.

It should also be noted that the urban–rural differences in the association between social participation and remaining teeth exist under potential influencing factors such as living environment and social-participation patterns in China. For urban older adults, promoting social participation (formal and informal) seems to be an important aspect of public-health policies which could decrease the risk of oral-health problem, such as tooth loss. However, for rural older adults, the priority lies in figuring out the status and changes in their social-participation pattern alongside social and economic development, and then how to encourage the positive role of social participation in their oral health promotion. In addition, the reasons and mechanism of the association between social participation and remaining teeth in urban and rural older adults may be different and need to be discussed and explored in future studies considering different social contexts.

## Figures and Tables

**Table 1:** Descriptive summary of study variables.

	Total (*n* = 11,948)	Urban (*n* = 6836)	Rural (*n* = 5112)	*p* Value
Gender, *N* (%)				<0.001
Male	5132 (43.0%)	3047 (44.6%)	2085 (40.8%)	
Female	6816 (57.0%)	3789 (55.4%)	3027 (59.2%)	
Age, mean (SD)	85.48 (11.87)	85.50 (11.82)	85.45 (11.94)	0.825
Household annual income, *N* (%)				<0.001
≤12,000	4003 (33.5%)	1767 (25.9%)	2236 (43.8%)	
12,000–55,000	3979 (33.3%)	2136 (31.2%)	1843 (36.0%)	
>55,000	3967 (33.2%)	2937 (42.9%)	1032 (20.1%)	
Education background				<0.001
Uneducated	5958 (49.9%)	2978 (43.6%)	2980 (58.3%)	
Primary school	3755 (31.4%)	2097 (30.7%)	1658 (32.4%)	
Middle school and high school	1805 (15.1%)	1356 (19.8%)	449 (8.8%)	
College or above	430 (3.6%)	405 (5.9%)	25 (0.5%)	
Marital status, *N* (%)				<0.05
Married and living with spouse	4736 (39.6%)	2765 (40.4%)	1971 (38.6%)	
Separated	204 (1.7%)	124 (1.8%)	80 (1.6%)	
Divorced	38 (0.3%)	28 (0.4%)	10 (0.2%)	
Widowed	6875 (57.5%)	3865 (56.5%)	3010 (58.9%)	
Never married	95 (0.8%)	54 (0.8%)	41 (0.8%)	
Smoking, *N* (%)				<0.01
Yes	1752 (14.7%)	946 (13.8%)	806 (15.8%)	
No	10,196 (85.3%)	5890 (86.2%)	4306 (84.2%)	
Alcohol drinking, *N* (%)				0.064
Yes	1658 (13.9%)	914 (13.4%)	744 (14.6%)	
No	10,290 (86.1%)	5922 (86.6%)	4368 (85.4%)	
BMI, *N* (%)				<0.001
Underweight	2533 (21.2%)	1381 (20.2%)	1152 (22.5%)	
Normal	6643 (55.6%)	3763 (55.0%)	2880 (56.3%)	
Overweight	2198 (18.4%)	1346 (19.7%)	852 (16.7%)	
Obesity	574 (4.8%)	347 (5.1%)	227 (4.5%)	
Sleep duration, *N* (%)				0.264
<7 h	6141 (51.4%)	3554 (52.0%)	2587 (50.6%)	
7–9 h	3381 (28.3%)	1922 (28.1%)	1459 (28.5%)	
>9 h	2425 (20.3%)	1358 (19.9%)	1067 (20.9%)	
Having NCD, *N* (%)				<0.001
Yes	3765 (68.5%)	1921 (28.1%)	1844 (36.1%)	
No	8183 (31.5%)	4915 (71.9%)	3268 (63.9%)	
ADL score, mean (SD)	7.28 (2.77)	7.39 (2.88)	7.13 (2.61)	<0.001
MMSE score, mean (SD)	23.02 (8.91)	23.64 (8.87)	22.16 (8.88)	<0.001
Informal social participation, *N* (%)				<0.001
Almost everyday	2958 (24.8%)	1528 (22.4%)	1430 (28.0%)	
Not daily, but once for a week	1822 (15.2%)	1030 (15.1%)	792 (15.5%)	
Not weekly, but at least once for a month	775 (6.5%)	439 (6.4%)	336 (6.6%)	
Not monthly, but sometimes	965 (8.1%)	596 (8.7%)	369 (7.2%)	
Never	5428 (45.4%)	3243 (47.4%)	2185 (42.7%)	
Formal participation, *N* (%)				<0.001
Almost everyday	322 (2.7%)	240 (3.5%)	82 (1.6%)	
Not daily, but once for a week	325 (2.7%)	246 (3.6%)	79 (1.5%)	
Not weekly, but at least once for a month	341 (2.9%)	261 (3.8%)	80 (1.6%)	
Not monthly, but sometimes	640 (5.4%)	441 (6.5%)	199 (3.9%)	
Never	10,320 (86.4%)	5648 (82.6%)	4672 (91.4%)	
Number of natural teeth, mean (SD)	9.48 (10.40)	10.09 (10.56)	8.65 (10.13)	<0.001

*p* values are calculated with analysis of Chi-squared test for categorical variables and T test for continuous variables.

**Table 2:** Multiple linear regression analysis of the association between social participation and remaining teeth.

	Model 1 (Crude OR)	Model 2 ^a^	Model 3 ^b^
Informal social participation	−1.142 *** (−1.271; −1.014)	−0.192 ** (−0.311; −0.074)	−0.152 * (−0.274; −0.030)
Formal social participation	−1.672 *** (−1.910; −1.434)	−0.404 *** (−0.619; −0.189)	−0.370 ** (−0.585; −0.156)

* *p* < 0.05; ** *p* < 0.01; *** *p* < 0.001; results are from proportional odds models. Results are displayed as regression coefficient and 95% confidence interval. ^a^ Adjusted for gender, age, education background, marital status, and household annual income. ^b^ Adjusted for gender, age, education background, marital status, household annual income, smoking, alcohol drinking, sleep duration, BMI, NCD, ADL, and cognitive function.

**Table 3:** Multiple linear regression analysis of the association between social participation and remaining teeth: urban vs rural.

	Urban			Rural		
	Model 1 (Crude OR)	Model 2 ^a^	Model 3 ^b^	Model 1 (Crude OR)	Model 2 ^a^	Model 3 ^b^
Informal social participation	−1.147 *** (−1.320; −0.974)	−0.285 *** (−0.444; −0.126)	−0.244 ** (−0.408; −0.081)	−1.232 *** (−1.425; −1.040)	−0.129 (−0.308; 0.051)	−0.080 (−0.266; 0.106)
Formal social participation	−1.697 *** (−1.979; −1.414)	−0.350 ** (−0.607; −0.093)	−0.309 * (−0.566; −0.052)	−1.155 *** (−1.627; −0.682)	−0.284 (−0.700; 0.132)	−0.311 (−0.727; 0.106)

* *p* < 0.05; ** *p* < 0.01; *** *p* < 0.001. Results are displayed as regression coefficient and 95% confidence interval. ^a^ Adjusted for gender, age, education background, marital status, and household annual income. ^b^ Adjusted for gender, age, education background, marital status, household annual income, smoking, alcohol drinking, sleep duration, BMI, NCD, ADL, and cognitive function.

## Data Availability

PKU Centre for Healthy Ageing and Development. 2021. “Chinese Longitudinal Healthy Longevity Survey (CLHLS)”. Peking University Open Research Data. https://doi.org/10.18170/DVN/WBO7LK (accessed on 18 October 2022).
